# Influence of diabetes and hypercholesterolemia on laboratory methods for hereditary spherocytosis diagnosis

**DOI:** 10.1002/jcla.24248

**Published:** 2022-01-26

**Authors:** Elena Lazarova, Béatrice Gulbis

**Affiliations:** ^1^ Laboratory of Hereditary RBC pathologies Department of Clinical Chemistry Laboratoire Hospitalier Universitaire de Bruxelles‐ Universitair laboratorium Brussel Université Libre de Bruxelles Bruxelles Belgium

**Keywords:** diabetes, diagnosis, hereditary spherocytosis, hypercholesterolemia, RBC deformability, red blood cells

## Abstract

**Introduction:**

Hereditary spherocytosis (HS) is characterized by decreased erythrocyte deformability resulting in hemolytic anemia. This is a heterogeneous disease regarding underlying protein deficiency, disease severity, age at diagnosis and clinical course. Although largely considered as pediatric disease, HS could be initially diagnosed also in elder patients as a result of gallstones or splenomegaly fortuitous finding. Concurrently, common adulthood metabolic disorders like diabetes or dyslipidemia are also known to impair RBC rheology and deformability. Therefore, we aimed to investigate if these diseases affect the screening and diagnostic tools used for HS diagnosis.

**Methods:**

We applied our workflow for HS diagnosis on 95 pathological samples: 29 patients with diabetes, 20 with hypercholesterolemia, 17 with dyslipidemia, 6 with hypertriglyceridemia, 23 with metabolic syndrome (MS). Thus, a total of 73 samples were analyzed by automated reticulocyte analysis, 52 by cryohemolysis test, and 41 by ektacytometry osmoscan analysis as we used two out of the three tests for each individual sample.

**Results:**

Applying our screening algorithm based on automated reticulocyte indices, a total of 4 samples (4.2%): one sample (5%) from the diabetes group and three samples (16.7%) from the MS group, positioned into the HS zone. However, no significant difference was found between any of the pathological groups and the controls for the cryohemolysis test or the osmoscan.

**Conclusion:**

While diabetes and hypercholesterolemia are pathologic conditions known to present with decreased erythrocyte deformability and disturbed rheology, their possible concomitant presence with HS would not interfere with the screening and confirmatory laboratory methods.

## Graphical abstract

Comparison of reticulocyte analysis parameters’ results (median ± interquartile range) between diabetes group, Diab (*n =* 20), dyslipidemia group, DL (*n =* 18), hypercholesterolemia group, Chol (*n =* 17), metabolic syndrome group, MS (*n =* 18), and control group (*n =* 76). Statistical difference: *p < 0.05. Applying our screening algorithm for hereditary spherocytosis (HS) diagnosis based on automated reticulocyte indices, a total of 4 samples (4.2%): one sample (5%) from the diabetes group and three samples (16.7%) from the metabolic syndrome group, positioned into the HS zone. However, no significant difference was found between any of the pathological groups and the controls for the cryohemolysis test or the ektacytometry osmoscan. While diabetes and hypercholesterolemia are pathologic conditions known to present with decreased erythrocyte deformability and disturbed rheology, their possible concomitant presence with HS would not interfere with the screening and confirmatory laboratory methods.

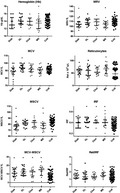

## 1 INTRODUCTION

Hereditary spherocytosis (HS) is the most frequent membranopathy and congenital hemolytic anemia in Northern Europe.^1^, ^2^ Typical HS presentation includes general signs of hemolysis combined with characteristic RBC morphology that is, spherocytes, family history, altered MCHC, and automated reticulocyte parameters that is, mean reticulocyte volume (MRV), mean sphered cell volume (MSCV), and immature reticulocyte fraction (IRF).^2^, ^3^ Novel screening algorithms were proposed, based on easily available and rapid automated hematological parameters such as delta (MCV‐MSCV)^4^ and MRV,^5^, ^6^ as well as IRF isolated or in a ratio to reticulocyte count.^7^ These achievements were included in the latest published guidelines for RBC membrane disorders diagnosis.^3^ More recent studies confirmed these findings and underlined the place of reticulocyte automated parameters in HS screening.^8^, ^9^

Specialized tests, reviewed by Bolton‐Maggs et al.^2^ and by King et.al.^3^ are generally recommended to confirm the diagnosis of HS for example, osmotic fragility test, cryohemolysis test, acidified glycerol lysis test, and eosin 5‐maleimide (EMA) binding test. In order to improve their predictive values, the combination of minimum two screening tests has been recommended.^10^ Further, another diagnostic tool for HS and other membranopathies, introduced several decades ago,^11^ is the osmotic gradient ektacytometry. Its importance was reassessed in the latest guidelines for RBC membrane disorders diagnosis,^3^ underlining its ability to differentiate various membranopathies. Approximately at the same period, a next generation ektacytometer was introduced: Laser‐assisted Optical Rotational Cell Analyzer, LoRRca, RR Mechatronics, Hoorn, the Netherlands.^12^ Its clinical implementation was first reported by Da Costa et al.^13^, followed by our group.^14^ Next generation ektacytometry (osmoscan) is currently a recognized method for RBC membranopathies diagnosis as an efficient intermediate step between screening tests, that is, RBC morphology, automated reticulocyte parameters, cryohemolysis, and EMA‐5 binding, and confirmatory protein deficiency tests, that is, sodium dodecyl sulfate polyacrylamide gel electrophoresis or DNA‐based methods.^14^, ^15^, ^16^, ^17^

The main underlying pathophysiological feature of HS is the deteriorated RBC mechanics and decreased cellular deformability based on structurally modified red cell membrane, decreased surface to volume ratio, and loss of elastic modulus with resultant hemolytic anemia.^18^ HS is a heterogeneous disease regarding protein deficiency, disease severity, age at diagnosis, and clinical course.^2^, ^18^ Although largely considered as pediatric disease, HS could be also initially diagnosed in adults and in elder patients as a result of, for example, gallstones or splenomegaly fortuitous finding.^18^ Moreover, common adulthood metabolic disorders like diabetes or dyslipidemia, are known to likewise impair RBC rheology and deformability. Various studies, reviewed recently,^19^, ^20^ have demonstrated association of increased plasma and whole blood viscosity with abnormal RBC membrane structure and intracellular viscosity in diabetes, leading to reduced RBC cell deformability and vascular complications such as diabetic microangiopathic nephropathy and retinopathy. Furthermore, these conditions were reported to be associated with anisocytosis, reflected by increased red distribution width, RDW.^21^

Measurement of RBC deformability is performed by various techniques that could be divided in two groups: (a) individual cell measurements for example, micropipette aspiration, atomic force microscopy, optical tweezers or (b) measurements of multiple cells for example, filtration method, microfluidic filtration, laser diffractometry (ektacytometry).^19^ To characterize RBC deformability, one mode is largely in use for membranopathies diagnosis: osmotic gradient ektacytometry. Shear stress (SS) is maintained constant while RBCs are mixed in the medium with gradually increasing osmolality, from 50 to 500 mOsm/kg. Measured diffraction pattern is subsequently a function of osmolality.^11^ It allows evaluation of the erythrocyte response to various factors affecting their cell geometry, internal viscosity, or membrane deformability.^13^ The ektacytometry has become one of the most applied methods in the field of RBC deformability study due to its accuracy, sensitivity, and convenience.^22^

Using osmotic gradient ektacytometry, a slight decrease of RBC deformability of the studied diabetic subjects, together with increased membrane protein glycosylation and oxidatively damaged spectrin, was demonstrated.^23^ However, in other studies, rheological behavior of the oldest and youngest 10% fractions of diabetic RBC was examined by classical rheoscope analysis, and it was found to be identical to that of normal cells.^24^ Besides, no decreased erythrocyte deformability was found either by filtration or by ektacytometry.^25^ Though, impaired deformability of diabetic RBC was demonstrated later by different deformability studies based on filtration and micropipette techniques and on SS diffractometer measurement by Rheodyn SSD (Myrenne GmbH, Roetgen, Germany).^26^ Succeeding, a disposable microfluidic ektacytometer, RheoScan‐D, (RheoMeditech)^27^ combining laser diffraction system and slit rheometer has been introduced for RBC deformability measurement in terms of elongation index (EI) which assessment was in agreement with that measured by conventional ektacytometer.^27^ Also, with this equipment, reduced RBC deformability was observed in diabetes, and the EI was even further decreased in diabetic patients with microvascular complications.^28^ Furthermore, decreased deformability manifested by increased osmotic fragility and additional membrane protein modifications, that is, a significant quantitative reduction in ankyrin, affecting the cytoskeleton rigidity and cell fragility, and plasma protein glycation and lipid peroxidation were also reported in diabetic RBC.^29^, ^30^

Another common adult metabolic disorder, hypercholesterolemia, a cause of accelerated atherosclerosis, is characterized by increased blood cholesterol. Indirect result is disturbed blood rheology: blood viscosity, platelet activation, and RBC deformability may be affected by hypercholesterolemia.^31^ Additionally, the same study demonstrated that long‐term cholesterol‐lowering therapy improved impaired RBC deformability, measured under a weak SS by laser diffractometric ektacytometer. Thus, it was hypothesized that improved RBC deformability may on its turn have beneficial effect on organ perfusion, including microcirculatory coronary blood flow.^31^ It should be noted that analyzing erythrocyte deformability under a high SS of 23.6 dyn/cm^2^ and greater gave no difference for hypercholesterolemia patients compared to control subjects.^31^ In another study, EI was measured in familial hypercholesterolemia adult group without treatment, using Rheodyn SSD, at different SS ranging from 0.30 to 60 Pa.^32^ Lower EI values were demonstrated for hypercholesterolemia patients at high SS >6 Pa; however, no differences were observed between patients and controls at lower SS. In experimental in vitro conditions, the relationship between cholesterol and ATP release from RBCs was studied with a microfluidic flow‐focusing device.^33^ It was found that decreasing membrane cholesterol increased cell deformability and ATP release; moreover, RBC deformability and ATP release were improved by simvastatin treatment by its direct membrane action of cholesterol enrichment.^33^

On the one hand, older patients are often affected by common medical conditions like diabetes or dyslipidemia, known to be characterized, inter alia, by hemorheological alterations and decreased RBC deformability. On the other hand, mild forms of HS could be suspected and diagnosed in adult or elder patient during family studies or investigation of accidentally found enlarged spleen or gallstones. As diabetes and hypercholesterolemia are pathologic conditions also known to disturb RBC deformability and rheology, we aimed to investigate if these diseases affect the screening and diagnostic tools used for membranopathies diagnosis. We focused on the screening tests already implemented in our laboratory, that is, automated reticulocyte parameters, cryohemolysis, and next‐generation ektacytometry with osmoscan and analyzed if there were interferences on these tests in the presence of hyperglycemia or hypercholesterolemia.

## 2 MATERIALS AND METHODS

### 2.1 Patients

Automated reticulocyte count, cryohemolysis test, and ektacytometry osmoscan analysis were performed during a three‐month period over a total of 95 samples from out‐clinic patients sent to the laboratory as part of their monitoring. Chronic diabetes patients were followed by our endocrinologist department. Chronic dyslipidemia patients were followed in our department of gastroenterology‐hepatology or in the department of vascular diseases. Samples were selected on daily basis with regard to the following criteria in order to avoid any bias related to vitamin or nutritional deficiency or concomitant inflammation on RBC besides the principal underlying condition: (a) chronic patients with at least one‐year follow‐up in our hospital and no acute disease episode at the moment; (b) no anemia, defined as Hb > 130 g/L for male patients and Hb > 120 g/L for female patents; (c) biological criteria for sample selection: 20 samples from patients with hypercholesterolemia (Chol): cholesterol >225 mg/dl without diabetes; 6 samples with isolated hypertriglyceridemia (TG), triglycerides >180 mg/dl without diabetes or hypercholesterolemia; 17 samples from patients presenting dyslipidemia (DL): defined as the combination of hypertriglyceridemia and hypercholesterolemia without diabetes; 29 samples from diabetic patients (Diab): glycated hemoglobin HbA1c >7.5% and/or fasting glucose >200 mg/dl without hypercholesterolemia or hypertriglyceridemia; 23 samples from patients with metabolic syndrome (MS): combination of diabetes and hypertriglyceridemia and/or hypercholesterolemia with the same triggers as above.

Control groups consisted of daily routine samples age‐ and sex‐matched to the study's population with hemoglobin, RBC, reticulocyte count, and biochemical parameters (ferritin, creatinine, fasting glucose, triglycerides, cholesterol, cardiac markers) in the reference ranges. The control group for the reticulocyte indices study consisted of 76 normal samples, for the cryohemolysis test it consisted of 6 normal samples run at the same time as the patient samples, and for the osmoscan analysis, it consisted of 14 normal samples run at the same time as the patient samples.

### 2.2 Assays

#### 2.2.1 DxH800‐automated reticulocyte analysis

UniCel DxH800 (Beckman Coulter) automates measured hemoglobin level, MCV, MRV, MSCV, delta (MCV‐MSCV), IRF (the count of reticulocytes with the highest RNA). The instruments were regularly controlled by internal and external quality controls, and their analytical variability was lower than 1% for all considered parameters.

#### 2.2.2 Cryohemolysis test

Cryohemolysis test was performed following Streichman et al.^34^ with minor adjustments as previously described.^5^

#### 2.2.3 LoRRca MaxSis ektacytometer and osmoscan curve

Osmotic gradient is generated by mixing low‐osmolar (40 mOsm/kg) and high osmolar (600 mOsm/kg) solutions, to which the blood sample is automatically and continuously mixed, as previously described.^14^ Briefly, RBC deformability, expressed as EI, is assessed and the obtained osmoscan curve is characterized by EI max (maximal EI), O min (the osmolality at EI min), O hyper (the osmolality in the hypertonic region at 50% of the EI max), and the area under the curve (AUC), spreading from O min to the hyper‐osmolar point of 500 mOsm/kg.

#### 2.2.4 Statistical analysis

Statistical analysis was realized by Analyse‐it Software, Ltd and GraphPad Prism software. Statistical significance for parameters with normal distribution was evaluated by unpaired *t* test; for parameters with non‐Gaussian distribution, Mann–Whitney *t* test and one‐way ANOVA Kruskal‐Wallis with one‐way analysis of variance (Dunn's multiple comparison test) were used; a level of *p* < 0.05 was considered as statistically significant.

## 3 RESULTS

A total of 95 individual pathological samples were included in the study. Generally, each sample was tested with 2 out of the three tests: reticulocyte count and cryohemolysis or osmoscan. A presentation of the total number of samples per group and per test is shown on Table 1. Because of the restricted number of patients in the group of hypertriglyceridemia, this group was merged in all following analysis to the dyslipidemia group.

**TABLE 1:** Number of samples per group and per test in the following groups: Diabetes, Dyslipidemia (DL), Hypercholesterolemia (Chol), Metabolic syndrome (MS), Hypertriglyceridemia (TG), and Controls

	Reticulocyte count	Cryohemolysis test	Osmoscan analysis
Diabetes	20	16	13
DL	14	10	7
Chol	17	10	10
MS	18	13	8
TG	4	3	3
Total	73	52	41
Controls	76	6	14

**FIGURE 1:**
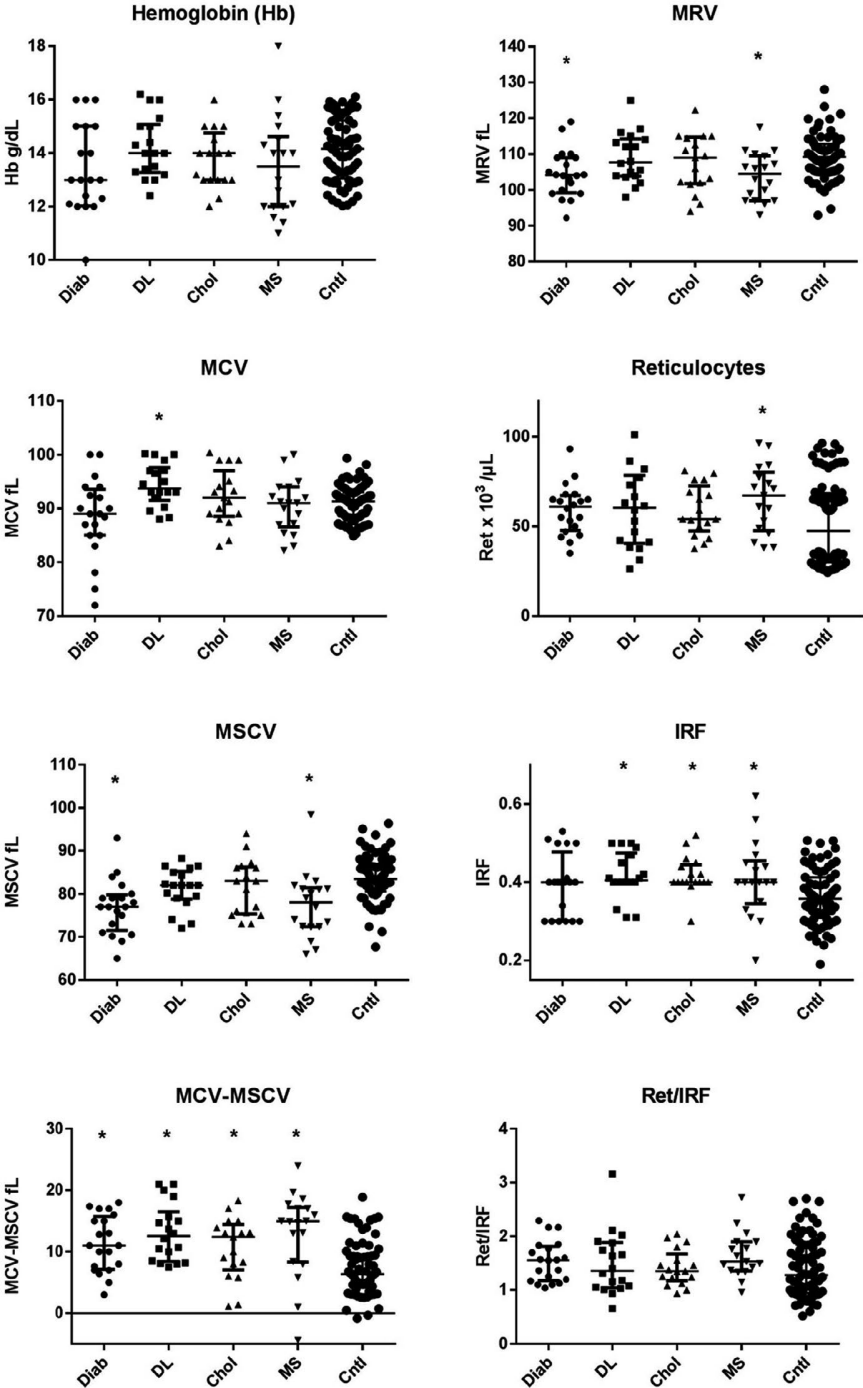
Comparison of reticulocyte analysis parameters’ results (median ± interquartile range) between diabetes group, Diab (*n* = 20), dyslipidemia group, DL (*n* = 18), hypercholesterolemia group, Chol (*n* = 17), metabolic syndrome group, MS (*n* = 18), and control group (*n* = 76). Statistical difference: *p < 0.05

When studying data obtained from the automated hematological analyzer, several statistical differences were found (Table 2 and Figure 1). There were no differences for hemoglobin level as patients presented no anemia following the selection criteria. However, MCV was significantly higher in the dyslipidemia group, MSCV was significantly lower in diabetic and MS groups, which explains why delta (MCV‐MSCV) was higher for all pathological groups. Regarding reticulocyte number and their indices, only in MS group we observed slight elevation in the reticulocyte count, still remaining in the reference range. MRV was significantly lower in diabetes and MS groups and IRF showed statistical differences for several groups. However, Ret/IRF ratio showed no difference for any of the pathological groups compared to the control group.

**TABLE 2:** Hemoglobin and automated erythrocyte and reticulocyte parameters in diabetes group, Diab (*n* = 20), dyslipidemia group, DL (*n* = 18), hypercholesterolemia group, Chol (*n* = 17), metabolic syndrome group, MS (*n* = 18), and control group (*n* = 76)

Parameter	Diab	DL	Chol	MS	Cntl
Hemoglobin (g/L)	130 (90–160)	140 (124–162)	140 (120–160)	135 (110–180)	142 (120–160)
MCV (fl)	89 (72–100)	94* (88–100)	92 (83–100)	91 (82–100)	91 (85–99)
MSCV (fl)	77* (65–93)	82 (72–88)	83 (73–94)	78* (66–98)	83 (68–96)
MCV‐MSCV (fl)	11* (3–18)	12.5* (7.5–21)	12.4* (1–18.3)	14.9* (−4–24)	6.3 (−0.8–19)
MRV (fl)	104.2* (92.2–119)	107.7 (98–125)	109 (94–122)	104.5* (93–117.5)	109.2 (93–128)
Ret (×10^9^/L)	61 (35–93)	60.4 (26–158)	54 (37–81)	67* (38–96)	47 (24–96)
IRF	0.4 (0.3–0.5)	0.4* (0.3–0.5)	0.4* (0.3–0.5)	0.4* (0.2–0.6)	0.35 (0.2–0.5)
Ret/IRF	1.6 (1–2.3)	1.4 (0.7–3.2)	1.4 (0.9–2)	1.6 (1–2.7)	1.3 (0.5–2.7)

Note

Results are expressed as median (minimum‐maximum). *Statistically significant difference between the pathology group and the control group, *p *< 0.05.

Applying our screening algorithm with defined cut‐offs for HS screening,^5^ that is, MSCV <70.2 fl and delta (MCV‐MSCV) >10.4 fl, one sample (5%) from the diabetes group and three samples (16.7%) from the MS group fell into the HS zone. All four samples suspected therefore for HS showed additionally MRV <96.7 fl, confirming thus the HS cautiousness. On the contrary, no sample from the hypercholesterolemia or from the dyslipidemia groups could be assumed as positively screened for HS.

**FIGURE 2:**
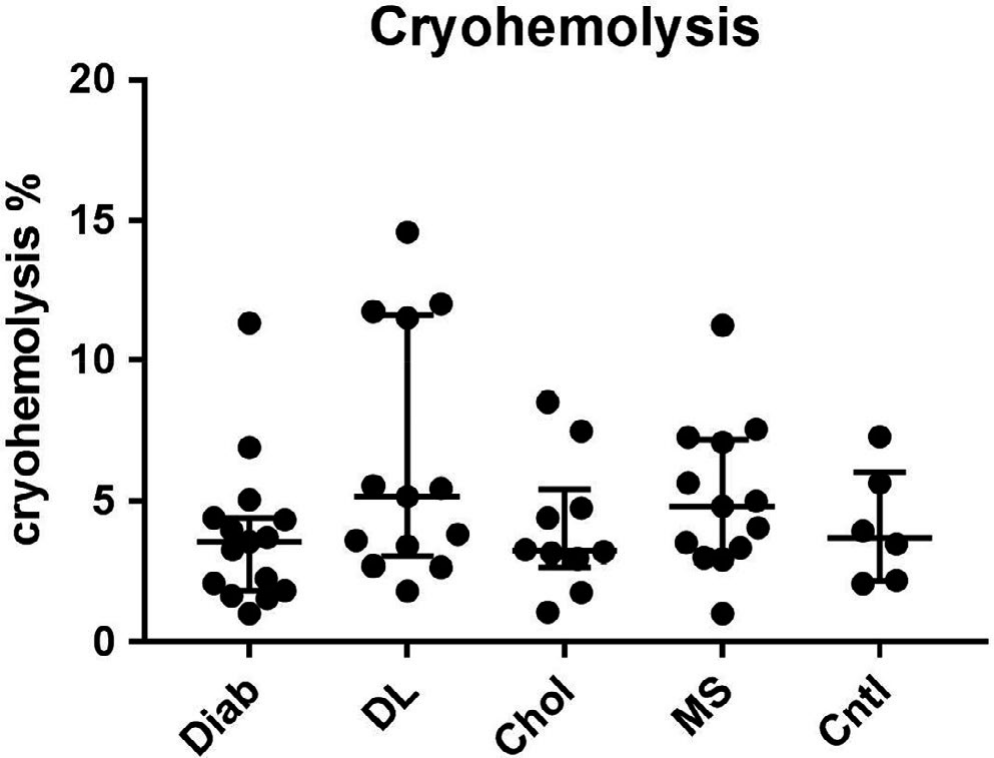
Comparison of cryohemolysis test result (median ± interquartile range) between diabetes group, Diab (*n* = 16), dyslipidemia group, DL (*n* = 13), hypercholesterolemia group, Chol (*n* = 10), metabolic syndrome group, MS (*n* = 13), and control group (*n* = 6)

Observing the cryohemolysis test, no significant difference was established between different pathology groups and the control group (Figure 2).

**FIGURE 3:**
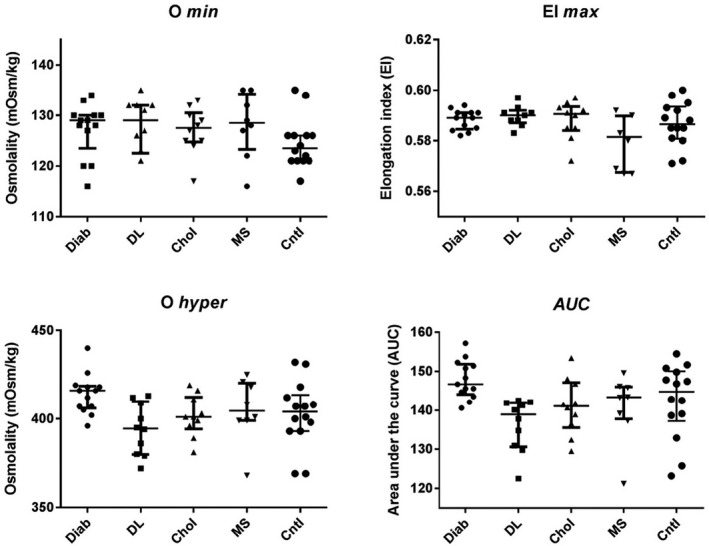
Comparison of osmoscan curve parameters (median ± interquartile range) between diabetes group, Diab (*n* = 13), dyslipidemia group, DL (*n* = 10), hypercholesterolemia group, Chol (*n* = 10), metabolic syndrome group, MS (*n* = 8), and control group (*n* = 14)

With regard to the parameters of clinical interest of the osmoscan curve, that is, O min, O hyper, EI max, and AUC, none showed statistically significant difference between the different pathology groups and the control group (Figure 3).

## 4 DISCUSSION

With the present study, we aimed to analyze if common pathologic conditions such as diabetes or hypercholesterolemia interfere with the panel of screening tests for HS implemented in our laboratory practice.

There are few published studies investigating the effects of hyperglycemia^21^, ^35^ and hypercholesterolemia^36^ on RBC blood indices; they focused only on erythrocyte indices such as RDW, MCV, and MCH and gave contradictory results concerning specifically MCV in dyslipidemia.

Regarding our data obtained with the automated hematological analyzer, several parameters presented moderate increase or decrease compared to the control group (Table 2 and Figure 1). It is important to be noted that all results remain in the reference ranges established in our laboratory although moderate statistical differences were found regarding MSCV, delta (MCV‐MSCV), and MRV between pathological groups and controls. Applying our screening algorithm,^5^ no sample from the hypercholesterolemia or from the dyslipidemia groups could be assumed positively screened for HS. Though, one sample from the diabetic and three samples from the MS group could have been suspected for HS, demonstrating findings of lower MSCV and MRV similar to HS. However, these samples showed normal results with the cryohemolysis test or with the osmoscan analysis, thus HS could be overruled. In fact, MSCV is an artificial volume reflecting mild swelling induced by the hypo‐osmotic solution used for spherization before reticulocyte count measurement.^37^ It is possible that sphered RBC from diabetic and MS patients reach their critical volume faster in hypo‐osmotic conditions and partial fragmentation occurs more intensely.

It was demonstrated that hyperglycemia resulted in diverse modifications of the RBC membrane and cytoplasm, which could disturb RBC functionalities, comprising deformability.^19^ Surface–volume ratio, internal viscosity, and dynamic membrane properties are main determinants of RBC deformability. Several typical for diabetes alterations affect these factors^26^: variations of cytoplasmic sorbitol concentration and membrane cholesterol impact the surface–volume ratio; increased Ca2+ or HbA1c concentrations or decreased organic phosphates may alter the cellular viscosity; variations in cholesterol–phospholipid ratio, fatty acid saturation and membrane proteins influence the membrane dynamic properties.^26^ With regard to hypercholesterolemia, RBC deformability was found decreased, conceivably as a consequence of modifications in lipid membrane composition and in ATP release intensity.^33^ However, evaluation of RBC deformability is challenging, due to the absence of standardized techniques. Furthermore, the observed discrepancies between various studies may be explained by differences in the studied populations or in the methodologies used to measure RBC deformability.^26^

Cryohemolysis test^34^ is based on the measurement of hemolysis of washed RBC diluted in hypertonic solution at 37°C and then subjected to lower temperature (0°C) provoking lipid phase transition and massive hemolysis. The range of cryohemolysis in normal subjects is 3%–15%; in HS, it is >20%.^38^ It was also demonstrated that artificially provoked spherocytes, which are known to be very fragile osmotically, presented with normal cryohemolysis.^34^ This suggests that surface–volume ratio reduction alone does not affect the cryohemolysis level. It was postulated that the straining inflicted on the cytoskeleton during the temperature changes exposed defects in the proteins participating in the interskeletal vertical interactions. Thus, the cryohemolysis test depends on the properties of the erythrocyte membrane related to the disturbed intimate binding of skeletal proteins to membrane phospholipids.^38^

To our knowledge, there are no studies published over cryohemolysis test in diabetic or dyslipidemia patients. Our data (Figure 2) demonstrate no differences between pathological samples and control samples, all pathologic samples giving a result of the cryohemolysis test in the reference range <15%, undoubtedly indicating no interference of these pathologies with HS screening by cryohemolysis test. Although a significant reduction in the quantity of the ankyrin protein was shown in diabetic patients,^29^ our data indirectly prove that the underlying modifications of the RBC membrane in diabetes do not affect the vertical interskeletal interactions contrary to HS. Apparently, diabetic and hypercholesterolemic RBC are capable of coping with hypertonic solution and then with the membrane changes upon cooling.

With regard to the parameters of clinical interest of the osmoscan curve, that is, O min, O hyper, EI max, and AUC, none of them showed statistically significant difference between the different pathology groups and the control group (Figure 3). We used in our study the osmoscan modus of LoRRca as this is the one actually largely used in hereditary membranopathies diagnosis.^14^, ^15^, ^16^, ^17^ To our knowledge, this study is the first one analyzing diabetic and dyslipidemia samples by osmotic gradient ektacytometry performed by LoRRca.

Decreased deformability was previously observed in 66% of the studied diabetic population by conventional osmotic gradient ektacytometry, analyzed by osmotic gradient ektacytometry (0–500 mOsm) under constant SS (120 dyn/cm^2^ or 12 Pa) and by iso‐osmotic osmoscan at 290 mOsm under SS gradient (0–212 dyn/cm^2^).^23^ However, these studies were performed at lower SS than LoRRca, 12 Pa versus 30 Pa, which could explain the controversy with our findings. Unfortunately, additional parameters of the osmoscan curve, that is, O min, O hyper, and AUC were not reported. Indeed, LoRRca analyzes in a principle similar to classical ektacytometry although the way of generating the deformability index is different, consequently is the value of EI max.^12^ Nevertheless, it was confirmed by a comparative study between LoRRca and conventional ektacytometer^39^ that, though different values of EI max are provided, the ektacytometry curves present extremely similar shapes. As routine patients’ curves are always compared to the normal control curve, there is no considerable amplitude difference.

Regrettably, no other authors, to our knowledge, published additional data about diabetic or dyslipidemia patients regarding osmotic gradient ektacytometry neither with the classical ektacytometers nor with the new‐generation one. Most of the following studies based on ektacytometry measurements investigated RBC deformability only in the mode of increasing SS applied to constant mixture osmolality. A study conducted with the precursor of LoRRca, in which the osmoscan modus was absent, studied deformability in four groups of diabetic patients: without organ complication or with microalbuminuria, nephropathy, and leg ulceration.^25^ Deformability was assessed by EI measurements under 30 Pa SS in normo‐osmolar (300 mOsm/kg) and in hyper‐osmolar solutions (400 mOsm/kg) in order to imitate local environment of the peritubular kidney capillaries; no difference was found between diabetic patients and controls. Furthermore, there was no correlation between RBC deformability and plasma glucose or HbA1c levels. Because the maximum EI measured at 30 Pa in normo‐osmolar solution is directly related to EI max of the osmoscan curve, not surprisingly, our data, showing no difference between diabetic (and dyslipidemic) osmoscan curves and normal controls, are in agreement with the findings of Schut et al.^25^

Presently, several ektacytometers, based on the same laser‐diffraction principle but applying different shearing geometries (e.g., Couette, plate–plate, microchannel) are commercialized. Measured EI is reported by all instruments as a range of SS; SS range is user selectable for some devices.^22^ The International Society for Clinical Hemorheology compared three ektacytometers: LoRRca, Rheodyn SSD, and RheoScan‐D. Intra‐assay variations for SS >1 Pa were below 5% for all instruments although higher variations were found for Rheodyn SSD and RheoScan‐D at lower SS; all ektacytometers detected the effects of glutaraldehyde or heat treatments.^22^ RheoScan‐D measures under a smaller SS range (0.5–20 Pa) than LORCA or Rheodyn SSD (0.35–75 Pa). Nevertheless, measurement range difference does not affect RheoScan‐D value because all three instruments possess their most useful SS region for detecting differences with best sensitivity.^22^

All studies demonstrating decreased deformability of RBC in diabetes^26^, ^28^, ^40^, ^41^, ^42^ or in hypercholesterolemia,^31^, ^32^ using RheoScan‐D^28^, ^42^ or Rheodyn SSD^26^, ^32^, ^40^, ^41^ or a similar laser diffractometric method,^31^ were based on measuring EI at lower SS than LoRRca in its osmoscan modus does. The discrepancy observed between our study and the previous studies could be explained by the distinctive instruments and specifically by the differences in the applied SS. However, the aim of our study was to investigate if there were interferences by diabetes or hypercholesterolemia in osmoscan used for HS diagnosis. Obviously, these common adulthood pathologies would not potentially interfere with a concomitantly present membranopathy under LoRRca osmoscan mode.

In conclusion, even if diabetes and hypercholesterolemia are pathologic conditions known to present with reduced RBC deformability and rheology, their potential concomitant occurrence with HS would not interfere with the laboratory diagnostic methods.

## CONFLICT OF INTEREST

The authors declare no conflict of interest.

## Data Availability

The data that support the findings of this study are available from the corresponding author upon reasonable request.
